# Diabetes Insipidus as a Rare Complication of Metastatic Breast Cancer With Pituitary Metastases

**DOI:** 10.7759/cureus.115755

**Published:** 2026-09-04

**Authors:** Francis T Mutamba, Mohammed Aamir, Kingsley C Okpii, Lakshmi Balakrishnan, Pooja Nanwani

**Affiliations:** 1 Diabetes and Endocrinology, University Hospitals Birmingham NHS Foundation Trust, Birmingham, GBR; 2 Haematology, University Hospitals of Leicester NHS Trust, Leicester, GBR; 3 Oncology, University Hospitals of Leicester NHS Trust, Leicester, GBR; 4 Stroke Medicine, University Hospitals Birmingham NHS Foundation Trust, Birmingham, GBR

**Keywords:** central diabetes insipidus (cdi), metastatic breast cancer (mbc), pituitary metastasis, polydipsia and polyuria, posterior pituitary metastases

## Abstract

Diabetes insipidus (DI) is a rare but significant and life-threatening complication of metastatic breast cancer, typically arising when cancer spreads to the pituitary gland. This condition results from impaired secretion or action of antidiuretic hormone (ADH) or arginine vasopressin (AVP), leading to symptoms including excessive urination and thirst. The posterior lobe of the pituitary and the infundibulum are most susceptible to metastatic involvement due to their direct arterial blood supply, making them more accessible to circulating tumor cells. This phenomenon explains why DI can be a manifestation of pituitary metastases. We report the case of a patient who presented with a five-week history of projectile vomiting, dizziness, blurred vision, headache, insatiable thirst, and nocturia. She reported urinating up to 12 times at night such that she struggled to sleep and would end up having daytime lethargy and fatigue. Biochemical evaluation demonstrated hyperosmolar serum, low urine osmolality, and low urine sodium, consistent with central DI. MRI revealed a pituitary stalk and suprasellar metastases with multiple new cerebral and cerebellar lesions. The patient underwent a desmopressin challenge leading to rapid improvement in polyuria and an increase in urine osmolality. Desmopressin confirmed an intact renal response, establishing the diagnosis of cranial DI secondary to pituitary metastases. Despite symptomatic benefit, her overall condition deteriorated due to progressive intracranial and systemic disease. She subsequently received whole-brain radiotherapy and palliative care but died three months after the diagnosis of DI. This case highlights central DI as a rare but clinically important manifestation of pituitary metastases in metastatic breast cancer. Prompt recognition and endocrine replacement, particularly with desmopressin, can provide meaningful symptomatic relief even when prognosis remains poor. Early multidisciplinary involvement, including endocrinology, oncology, radiology, and palliative care, is essential for optimizing quality of life in such complex cases.

## Introduction

Diabetes insipidus (DI) is an uncommon but clinically significant complication of advanced malignancy, most often associated with pituitary metastases [1]. It arises from impaired secretion or action of antidiuretic hormone (ADH), also called arginine vasopressin (AVP), leading to profound polyuria, polydipsia, and risk of severe electrolyte imbalance. Pituitary metastases themselves are rare, accounting for approximately 1% of all pituitary tumors [2], with breast and lung cancers representing the most frequent primary sources [3,4]. The posterior pituitary and infundibulum are particularly vulnerable due to their direct arterial blood supply, which facilitates tumor cell dissemination [5]. Consequently, central DI is the most common endocrine manifestation of pituitary metastases [6]. Although rare, the recognition of DI in the context of metastatic breast cancer is critical, as it often signifies advanced disease and carries a poor prognosis [7]. Prompt diagnosis and initiation of targeted endocrine therapy, particularly desmopressin, can provide significant symptomatic relief and improve quality of life, even when curative treatment is no longer feasible.

## Case presentation

This is a case of a woman with a background of breast cancer who presented with a five-week history of recurrent episodes of projectile vomiting, which were associated with persistent dizziness and progressively worsening blurred vision. Over time, she began experiencing severe headaches, described as dull and continuous, which often interfered with her daily activities. The most striking complaint was an insatiable thirst accompanied by marked polyuria and nocturia. She reported drinking large volumes of water throughout the day yet continued to experience unrelenting thirst. At night, she was forced to wake up to urinate up to 12 times, producing large volumes of dilute urine “like water.” This severely disrupted her sleep, leading to significant daytime lethargy, fatigue, and reduced ability to perform activities of daily living. She also noted a modest but noticeable weight loss during this period, which she attributed to vomiting and excessive urination. Importantly, she denied fevers, cough, dysuria, or classic symptoms of diabetes mellitus such as polyphagia or weight loss. Her visual symptoms were particularly concerning. She described progressively worsening blurred vision, often associated with dizziness and headaches. These symptoms raised concern for an intracranial process.

Review of systems

The patient reported no cardiovascular symptoms, including palpitations, chest pain, shortness of breath, or peripheral edema. There was no history of cough or hemoptysis. Neurologically, she reported no seizures, limb weakness, sensory loss, or episodes of confusion. She had no gastrointestinal symptoms, including abdominal pain, diarrhoea, or constipation, and her bowels were opening normally. Her past medical history was unremarkable apart from triple-negative breast cancer. She had previously lived independently but, due to the severity of her symptoms, had moved in with her mother for support. She was an ex-smoker, with a previous history of smoking six cigarettes per day, and denied alcohol or illicit drug use. Her family history was non-contributory.

On arrival, her Early Warning Score was 0. She was alert, stable, and afebrile, with a Glasgow Coma Scale score of 15/15. General inspection revealed dry mucous membranes and skin, consistent with mild dehydration.

Her cardiovascular examination revealed a regular pulse, normal heart sounds, no murmurs, and no raised jugular venous pressure. Respiratory examination revealed normal breath sounds bilaterally, good air entry, and no crepitations. Neurological examination showed intact cranial nerves. Motor examination revealed normal tone, reflexes, sensation, and full power (5/5) in both upper and lower limbs.

Laboratory investigations at diagnosis are included in Table 1.

**Table 1:** Laboratory Investigations at Presentation

Test	Result	Reference Range (approx.)
C-reactive Protein	<5 mg/L	<10 mg/L
Full Blood Count		
White Cell Count	11.0 x10^9/L	4.0–11.0 x10^9/L
Hemoglobin	93 g/L	120–160 g/L
Platelets	356 x10^9/L	150–400 x10^9/L
Neutrophils	9.74 x10^9/L	2.0–7.5 x10^9/L
Urea and Electrolytes		
Sodium	140 mmol/L	135–145 mmol/L
Potassium	4.1 mmol/L	3.5–5.0 mmol/L
Urea	10.4 mmol/L	2.5–7.5 mmol/L
Creatinine	87 µmol/L	45–90 µmol/L
Estimated Glomerular Filtration Rate	66 mL/min/1.73m²	>90 mL/min/1.73m²
Liver Function Tests		
Alanine Transaminase	43 U/L	7–56 U/L
Total Bilirubin	20 µmol/L	5–21 µmol/L
Total Protein	67 g/L	60–80 g/L
Albumin	42 g/L	35–50 g/L
Alkaline Phosphatase	272 U/L	30–130 U/L
Coagulation Profile		
International Normalized Ratio	1.1	0.8–1.2
Prothrombin Time	14.4 seconds	11–15 seconds
Activated Partial Thromboplastin Time	24 seconds	25–35 seconds
Osmolality Investigations		
Serum Osmolality	303 mOsm/kg	275–295 mOsm/kg
Urine Osmolality	136 mOsm/kg	300–900 mOsm/kg (variable)
Urine Sodium	12 mmol/L	Variable, typically >20 mmol/L
Endocrine Panel		
Thyroid-Stimulating Hormone	0.21 mIU/L	0.4–4.0 mIU/L
Free Thyroxine	8.8 pmol/L	
		9–19 pmol/L
Follicle-Stimulating Hormone	1.4 IU/L	2–12 IU/L (varies by sex/age)
Luteinizing Hormone	<0.5 IU/L	2–12 IU/L (varies by sex/age)
HbA1c	4.3 %	4.0–5.6 %

MRI of the head (Figure 1) was performed, showing that the suprasellar, pituitary stalk, and hypothalamic metastatic lesion had significantly increased in size, with the suprasellar component measuring 16 × 20.5 mm. There was an increase in the size of the pituitary metastatic component, with new cystic changes within the pituitary lesion. New bilateral cerebral and cerebellar metastatic lesions were also noted. The patient was to be discussed at the pituitary multidisciplinary team (MDT).

**Figure 1:**
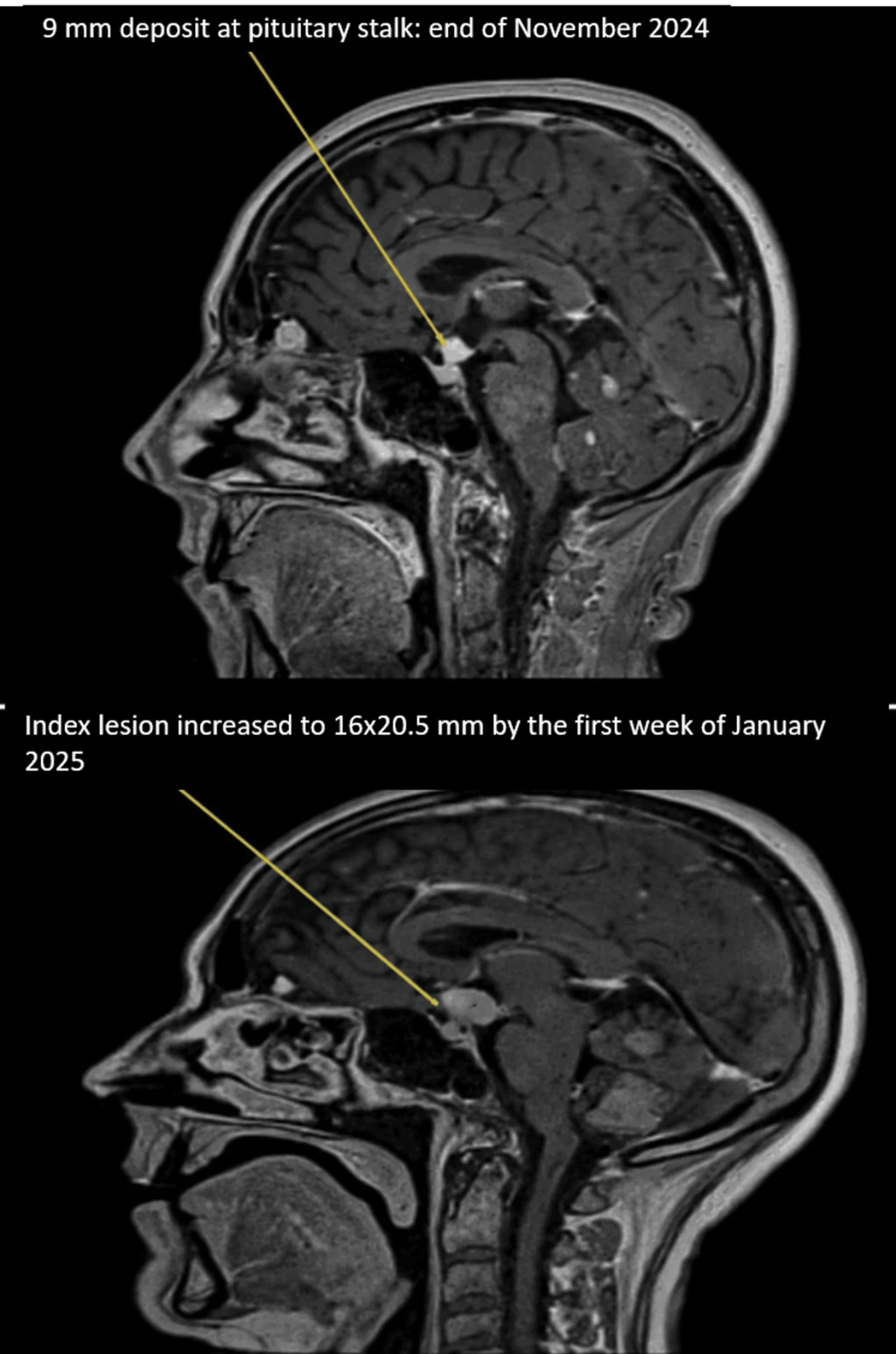
MRI of the brain brain (sagittal view) demonstrating progression of pituitary infundibular metastasis from breast cancer. Top: MRI obtained in end-November 2024 showed a 9-mm deposit at the pituitary stalk. Bottom: Follow-up MRI in early January 2025 demonstrated interval progression with enlargement of the index lesion to 16 × 20.5 mm, consistent with rapid metastatic infiltration of the pituitary infundibulum and suprasellar region.

The first differential diagnosis was cranial DI, which results from impaired synthesis or release of AVP from the hypothalamus, where it is produced, or the posterior pituitary, where it is stored and subsequently excreted. AVP works primarily in the kidneys, assisting with fluid and electrolyte balance and concentrating urine. Impaired synthesis or release of AVP leads to the kidneys’ inability to concentrate urine, leading to the patient presenting with the classic symptoms of polyuria, polydipsia, low urine osmolality, and hypernatremia. Our patient had low urine osmolality, with symptoms of polyuria and polydipsia with an insatiable thirst. She also had a urine output of more than four liters in 24 hours. The endocrinology team conducted a desmopressin trial; this led to an improvement in the symptoms of polyuria and polydipsia. This indicated an intact renal response and confirmed a central cause of the DI. Importantly, in this case, cranial DI is attributable to hypopituitarism resulting from metastatic breast cancer with pituitary gland involvement, as demonstrated by brain imaging showing metastatic lesions in the pituitary gland, which leads to infiltration and disruption of AVP-producing pathways. Hypopituitarism, whether autoimmune (hypophysitis) or secondary to metastases, is a known but rare cause of cranial DI. Breast cancer is among the malignancies with a predilection for metastasizing to the pituitary, and this secondary infiltration often presents with DI due to selective involvement of the posterior pituitary and infundibulum. The constellation of neuroimaging findings (MRI), clinical presentation, and response to desmopressin, along with the known diagnosis of metastatic breast cancer, strongly supports cranial DI secondary to metastatic hypopituitarism as the definitive diagnosis. Nephrogenic DI arises from renal resistance to AVP, typically due to congenital defects or acquired factors such as lithium therapy, hypercalcemia, chronic kidney disease, renal malignancy/metastases, and radiation exposure. In nephrogenic DI, urine osmolality fails to increase even after desmopressin, and polyuria persists, which distinguishes it from cranial DI. In this case, the patient demonstrated a marked improvement in polyuria after commencing desmopressin, ruling out renal AVP resistance. In addition, no history of nephrotoxic agents or electrolyte disturbances was identified to suggest an acquired cause. Primary polydipsia is defined as an excessive water intake due to a psychiatric illness or hypothalamic dysfunction. Consequent to this is a reduction of AVP, which then leads to urine dilution. Primary polydipsia’s presentation, although it mimics cranial DI, differs in the sense that the patient had an elevated serum osmolality and her urine output was significantly reduced on introducing AVP. We would expect only a modest reduction in urine output, if at all, if the patient had primary polydipsia because these patients already have normal endogenous AVP function and their kidneys are not AVP-deficient, so they are working normally. The excessive urination is due to excess water intake and not hormonal deficiency. A water deprivation test would have helped distinguish the two because in primary polydipsia there is an increase in urine concentration, while in cranial DI there is minimal increase in urine concentration. The water deprivation test was not done, unfortunately, in this context because with the underlying structural pituitary disease, primary polydipsia was not a plausible explanation. While primary polydipsia and nephrogenic DI were considered, the clinical presentation, biochemical findings, and the strong response to desmopressin, together with neuroimaging showing evidence of pituitary metastases on the background of metastatic breast cancer, confirm the diagnosis of cranial DI. This case shows that it is very important to consider central causes to certain symptoms in the context of metastatic breast cancer, as it is well known to metastasize to the brain. 

The patient was initially managed conservatively for suspected DI. Endocrine input was sought, and on review, they requested diagnostic investigations that revealed hyperosmolar serum with low urine osmolality and low urine sodium, which was consistent with central DI. An MRI brain scan showed multiple cerebral metastases, with a 9 mm lesion in the pituitary infundibulum, confirming the likely cause of AVP deficiency. The hormone profile noted secondary hypothyroidism and hypogonadotropic hypogonadism. This further confirmed hypopituitarism secondary to the infiltrative process by metastases. Initial management included oral levothyroxine 50 mcg once daily, oral dexamethasone 4 mg twice daily, and guidance to monitor urine output. Desmopressin was to be initiated if urine output exceeded 40 mL/kg/hr. On reassessment by the endocrine team, the patient was noted to have urine output >4 L/day; we commenced the patient on oral desmopressin 100 mcg twice daily. Polyuria improved within 48 hours, which confirmed the suspected diagnosis of cranial DI. Due to persistent vomiting and inability to tolerate oral medications, desmopressin was switched to a 10 mcg/dose intranasal spray twice daily, and dexamethasone was administered intravenously (3.3 mg twice daily). A repeat MRI revealed rapid progression of the pituitary stalk and suprasellar mass, now measuring 16 × 20.5 mm with new cystic changes and new bilateral cerebral and cerebellar metastases, prompting a referral to the pituitary MDT. The patient also received whole-brain radiotherapy during admission. She became progressively frail, and palliative care services were engaged; she was later discharged home for supportive care after one month of admission. Throughout the follow-up period of approximately three months from diagnosis of DI (end of December 2024) to death (mid-March 2025), the patient experienced significant decline in functional status, becoming increasingly bedbound with severe symptoms including back pain from vertebral compression fractures and episodes of anemia requiring multiple transfusions. She was unable to return to any normal daily activities or work and required comprehensive palliative care. This case underscores the importance of prompt diagnosis, multidisciplinary management, and integration of palliative care in patients with pituitary metastases and DI to optimize patient comfort and outcomes. 

## Discussion

Metastasis to the pituitary gland is a rare clinical occurrence, accounting for only 0.4%-0.87% of all intracranial metastases [8,9]. Pituitary metastases are notably uncommon compared to primary pituitary tumors, such as adenomas, which represent approximately 84.6% of pituitary lesions, while metastases comprise only 0.6% of cases [10]. The clinical presentation of pituitary metastasis can vary, but literature reviews indicate that the most frequent symptoms include visual disturbances (reported in 49%-62% of cases), panhypopituitarism (38%), and DI (28%-38%) [11,12]. However, a significant number of cases remain asymptomatic, complicating early detection and often leading to delayed diagnosis [11]. In the present case, the primary malignancy was breast cancer, one of the most frequently reported sources of pituitary metastases [11,13]. Currently, no standardized treatment guidelines exist for managing pituitary metastases [10]. Surgical intervention may offer symptomatic relief, particularly in cases of optic pathway compression, but it has not been shown to improve overall survival outcomes [9,13]. In contrast, systemic therapies such as radiotherapy and chemotherapy have shown a more favorable impact on survival [13]. However, chemotherapy was discontinued due to intolerable side effects. While previous studies have found no significant survival differences among various radiotherapy techniques, including conventional fractionated radiotherapy, Gamma Knife, and CyberKnife [14], some reports suggest that stereotactic radiotherapy may offer improved survival outcomes and symptomatic relief, including for DI and other neurological symptoms [13,15-17]. In this case, whole-brain radiotherapy was selected due to the presence of multiple brain metastases not amenable to stereotactic radiosurgery, as determined by the MDT. The reported mean survival following the diagnosis of pituitary metastasis ranges from 10 to 14 months [11,13]. More recent studies have shown improved survival rates compared to earlier data, likely reflecting advancements in neuroimaging, systemic therapies, and overall cancer care. This case highlights the importance of considering pituitary metastasis in patients with known malignancies who develop new endocrine or neurological symptoms. Early diagnosis and coordinated care involving a multidisciplinary team are essential to optimize patient outcomes in these rare but clinically significant cases.

## Conclusions

Central DI should be considered in patients with metastatic breast cancer presenting with polyuria, polydipsia, and electrolyte disturbances, especially when symptoms are disproportionate to known disease burden or suggest posterior pituitary involvement. Pituitary metastases, though rare, should be suspected in patients with known malignancy and new-onset endocrine dysfunction or neurological symptoms such as visual disturbances, vomiting, or altered thirst/urination patterns. A rapid and robust response to desmopressin therapy strongly supports a diagnosis of cranial (central) DI and helps distinguish it from nephrogenic DI and primary polydipsia. Prompt endocrine workup and initiation of hormone replacement (e.g., desmopressin, levothyroxine, dexamethasone) can significantly improve symptom control and patient comfort-even in the context of advanced metastatic disease. Early involvement of an MDT, including endocrinology, oncology, radiology, and palliative care, is essential for optimal management of complex cases like pituitary metastases with DI. 
